# Health-related quality of life among acute pancreatitis patients correlates with metabolic variables and associated factors

**DOI:** 10.1016/j.amsu.2022.104504

**Published:** 2022-09-06

**Authors:** Ojus Sardana, Pratima Kumari, Ravinder Singh, Hitesh Chopra, Talha Bin Emran

**Affiliations:** a Chitkara College of Pharmacy, Chitkara University, Punjab, India; b Department of Pharmacy, BGC Trust University Bangladesh, Chittagong, 4381, Bangladesh; c Department of Pharmacy, Faculty of Allied Health Sciences, Daffodil International University, Dhaka, 1207, Bangladesh

**Keywords:** Acute pancreatitis, Metabolic syndrome, Metabolic variables, Short form survey, 36

## Abstract

**Introduction:**

Acute pancreatitis (AP) and associated metabolic abnormalities constitute prevalent medical disorders that have disastrous implications and expensive cost of care. However, the connection with metabolic abnormalities and their influence on wellbeing i.e., health-related quality of life (HRQoL) remains unclear. As a result, we investigated the influence of MetS components on HRQoL in AP patients.

**Methods:**

In a tertiary care hospital in North India, comprehensive observational research was undertaken with enrollment of subjects having AP along metabolic syndrome (MetS) or without was included. MetS was diagnosed for subjects using the National Cholesterol Education Program–Adult Treatment Panel III (NCEP-ATP III) guidelines. Various socio-demographic variables were also taken into consideration for the calculation of statistical significance (P ≤ 0.05) in AP patients. Student's *t*-test and Short Form-36 (SF-36) along with the association between AP and MetS, as well as their impact on HRQoL, was investigated finally with, Pearson Correlation Analysis Factor.

**Results:**

The study comprised 100 subjects or patients diseased of AP associated with MetS and 100 patients with AP associated without MetS. Gender, Age, Educational Status, Tobacco uses along with the metabolic variables were found to be statistically significant (P ≤ 0.05) and comparatively increased in patients with AP with MetSthan AP without MetS except HDL levels. Finally, a negative association between all metabolic variables with the exception of HDL, and AP was found to be producing deterioration in Health compartment scores.

**Conclusion:**

AP with MetS patients had a worse aggregate HRQOL than AP without MetS patients.

## Highlights

• Acute pancreatitis (AP) and associated metabolic abnormalities constitute prevalent medical disorders.
• We investigated the influence of MetS components on HRQoL in AP patients.
• The focus of this study is to figure out the relationship between MetS with quality of life among AP patients.
• AP with MetS patients had a worse aggregate HRQOL than AP without MetS patients.

## 1 Introduction

The growing global frequency and epidemic percentage of Acute Pancreatitis (AP) in several countries has posed a significant health burden in recent years. AP affects 34 people per 100,000 person-years globally, and the number of people afflicted is rising [1]. Approximately 20% of those who are afflicted acquire severe illness, resulting in substantial morbidity and death. In the recent decade, however, AP-related mortality has decreased to 0.8%. Improvements in prompt and accurate diagnosis, as well as care for extremely sick persons with AP, are most likely to be major reasons. However, death and long-term consequences remain significant [2,3]. Furthermore, AP may be linked to the development of a variety of metabolic problems, which can all be lumped together to form the Metabolic Syndrome (MetS) [4]. Obesity, hyperglycemia, dyslipidemia, and hypertension are all interconnected possible causes for MetS, which puts a person at risk for liver problems, heart disease, pancreatitis, and type 2 diabetes mellitus, among other things [5]. MetS is linked to an elevated risk of hospitalization and mortality; however dietary changes and novel medications might reverse metabolic abnormalities [6]. MetS prevalence ranged from 7% to 56% worldwide, with 18.3% to 35.8% in South Asia [7]. Increased urbanisation, current dietary habits, overweight, linked diabetes and related associated repercussions of MetS may all contribute to increased incidence of MetS [8]. AP and MetS, as well as the consequences of the illness load, have increased morbidity and lowered life expectancy in recent years, therefore assessing the impact of MetS on Health-Related Quality of Life (HRQOL) has received greater attention [9]. Poor HRQOL has apparent repercussions for diabetes, cardiovascular illness, and hepatic problems; however, the influence of MetS and AP on HRQOL is less studied and has yet to be firmly demonstrated [10,11].

In most research, obesity appears to have a deleterious impact which influence on the disease, albeit the connection with MetS and the seriousness of disorder is not known, also it has dearth of evidence on the topic [12]. Till now no Indian researchers employing the SF-36 to assess the influence of illness load on QOL in AP with and without MetS have been published. Furthermore, the increased incidence along with price of both in maintenance and curing create critical conditions in which further research is required [[13], [14], [15], [16]].

As a result, the focus of this study aimed to figure out the relationship between MetS with quality of life among AP patients in north India.

## 2 Methods

### 2.1 Study population, data collection and definitions

Patients with AP were studied in a tertiary care hospital in North India for a 6-month descriptive observational clinical research (June 2021 to November 2021) and were carefully examined to relate the effect of illness on wellness i.e., HRQoL in subjects with AP with MetS and without MetS and AP. Patients were diagnosed with AP and tested if two or more of the following criteria were present: abdominal discomfort, serum amylase and lipase levels two or more times normal, visualization technique (abdominal ultrasonography, computerized x-ray, or diagnostic radiology) [[17], [18], [19]].The checklist was used to gather and record several required characteristics such as sex, age, weight, height, and laboratory results. SPSS (Statistical Package for Social Sciences Version 21.0) software package was used to evaluate the value of significance (P) of socio-demographic factors and AP using the Fisher exact test. The metabolic risk variables were also examined using National Cholesterol Education Program–Adult Treatment Panel III (NCEP-ATP III criteria), which divided subjects into two groups: those with AP with MetS (n = 100) and those without MetS (n = 100). The age range of the patients selected for the observational study was 35–65 years of all sex. In-patient and out-patient acute pancreatitis patients were included along with the patients were able to give written informed consent and were able to comply with study procedures. According to this criterion, individuals must be diagnosed with at least three of the five components to consider them under criteria of MetS. The components are triglycerides (TGs) > 150 mg/dL, Waist circumference (WC) in males >102 cm and women >88 cm, blood pressure: systolic blood pressure SBP>130 mm Hg while diastolic blood pressure DBP>85 mm Hg, and fast blood glucose>150 mg/dL, high-density lipoprotein (HDL) cholesterol: <men 40 mg/dL; women <50 mg/dL [20]. Patients under the age of 35, those with a history of AP recurrence, pregnant or breastfeeding women, and those with pancreatic diseases (such as chronic pancreatitis, a pancreatic tumour, or a cyst) were all excluded from the trial. Patients were chosen solely on the basis of their diagnoses, with no knowledge of their SF-36 scores. As a result, our patient selection was neither random nor sequential. The health domains subscales were computed using responses to specific questions on the questionnaire. GraphPad prism version 8.4.3 was used to store the data and run the statistical analyses. The differences in all aspects of patients with AP with and without MetS were investigated using the Student's test for continuous variables. Several SF-36 categories were correlated with demographic and metabolic components in patients with AP with and without MetS using Pearson coefficient correlation. A difference of P ≤ 0.05 was judged significant. The study methodology, design, and consent forms were assessed and approved by the Chitkara University Institutional Human Ethical Committee and with Helsinki Ethics Declaration in Medical Research as revised in 2000.

### 2.2 Statistical analysis

A database was generated and descriptive statistical analysis performed in the SPSS (Statistical Package for Social Sciences) Version 21.0 and Graphpad prism version 8.4.3. For socio-demographic parameters association with subjects with MetS and without MetS Fisher Exact Test was used and Student's *t*-test was used for clinical and metabolic markers assessment and finally Pearson coefficient correlation was used to compare and correlate several SF-36 domains with patients suffering of AP without MetS and AP with MetS. Statistically significant was considered (P value) ≤ 0.05.

## 3 Results

The socio-demographics characteristics of study population (n = 200) from which (n = 100) were suffering with AP with MetS and (n = 100) were suffering with AP without MetS are shown in Table 1. Females suffering with AP with MetS were found to be more is number as compared to the males and the people greater than age of 50 were also more in number as compared to the people less than and equal to 50 suffering with AP with MetS and both the gender and age parameters were found to be statistically significant (P ≤ 0.05). Along with both of them educational status and tobacco use was also found to be statistically significant where people with no formal schooling and those on tobacco use were higher in number in those suffering with AP with MetS. The other demographics parameters that are area of residence and alcohol consumption were found not to be statistically significant.

**Table 1:** Factors associated with impact of socio-demographic parameters in acute pancreatitis without metabolic syndrome (MetS) and acute pancreatitis (AP) with MetS (Fisher exact test).

Patients' characteristics	Acute Pancreatitis		*P* Value
	Without MetS (n = 100)	With MetS (n = 100)	
Gender			
Female	28	64	**<0.001**
Male	72	36	
Age (in years)			
≤50	58	34	**<0.001**
>50	42	66	
Educational status			
No formal schooling	22	58	**0.028**
Primary (Grad 1-6)	12	18	
Secondary (Grad 7-12)	12	8	
Tertiary (Diploma/Graduation)	54	16	
Area of Residence			
Urban	62	74	0.095
Rural	38	26	
Tobacco use			
Yes	62	72	**0.003**
No	38	28	
Alcohol consumption			
Yes	70	76	0.06
No	30	24	

P value ≤ 0.05 was considered as statistically significant. *MetS Metabolic Syndrome*.

**Fig. 1:**
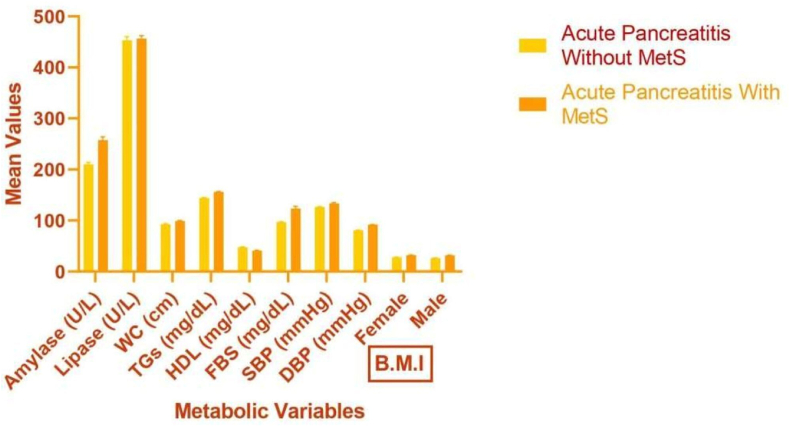
Mean values of patient's characteristics of patients with acute pancreatitis (AP) without and with metabolic syndrome (MetS). *B.M.I* body mass index*, DBP* diastolic blood pressure*, FBG* fasting blood glucose*, HDL* high-density lipoprotein*, MetS* Metabolic Syndrome, SBP systolic blood pressure, *TGs* triglycerides, *WC* waist circumference.

The clinical components of AP, such as Amylase and Lipase, as well as of metabolic disorders (WC, TGs, FBG, SBP, and DBP) were statistically significant in individuals with AP and MetS (P ≤ 0.05) and higher while HDL levels were also found to be statistically significant (P ≤ 0.05) but lower as compared to the other study group as shown in Table 2 and the corresponding Fig. 1. The difference in the BMI in males and females were also statistically significant (P ≤ 0.05) and were found to be higher in subjects with AP with metabolic disorders as compared to other groups.

**Table 2:** Illustration of mean scores of clinical variables of patients with acute pancreatitis (AP) without and with metabolic syndrome (MetS) (Student t-test).

Clinical Variables		Acute Pancreatitis		*P* Value
		Without MetS (n = 100)	With MetS (n = 100)	
Amylase (U/L)		210.04 ± 4.06	257.62 ± 6.43	**<0.001**
Lipase (U/L)		453.08 ± 7.28	456.48 ± 5.85	**0.041**
WC (cm)		92.6 ± 1.39	98.84 ± 1.39	**0.002**
TGs (mg/dL)		144.54 ± 0.72	155.84 ± 0.92	**<0.001**
HDL (mg/dL)		47.74 ± 0.95	41.06 ± 0.74	**<0.001**
FBG (mg/dL)		96.68 ± 1.03	123.5 ± 4.30	**<0.001**
SBP (mmHg)		126.52 ± 0.90	133.44 ± 1.60	**0.003**
DBP (mmHg)		80.98 ± 0.27	92 ± 0.49	**<0.001**
B.M.I	Female	27.90 ± 0.54	32.41 ± 0.32	**0.002**
	Male	26.50 ± 0.46	31.85 ± 0.33	**0.01**

Values are expressed as mean +standard error mean (SEM); P ≤ 0.05 was considered as statistically significant *B.M.I* body mass index*, DBP* diastolic blood pressure*, FBG* fasting blood glucose*, HDL* high-density lipoprotein*, MetS* Metabolic Syndrome, *TG* triglycerides, *WC* waist circumference.

A comparison of different HRQOL subscales in patients with and without MetS is illustrated in Table 3. The physical and mental aspects of AP with MetS were much lower as compared to those with AP without MetS. With the exception of BP and VT, all SF-36 domains (PF, RP, GH, SF, RE and MH) showed statistically significant higher (P ≤ 0.05) scores. The physical component summary (PCS) and mental component summary (MCS)scores derived were also found to be lower and statistically significant (P ≤ 0.05) in subjects suffering with AP with MetS as compared to another group.

**Table 3:** Illustration of mean scores of SF-36 domains in patients with acute pancreatitis with and without metabolic syndrome (Student t-test).

Health Values Short Forms 36 (SF-36)	Acute Pancreatitis		*P Value*
	Without MetS (n = 50)	With MetS (n = 50)	
PF	79.567 ± 1.51	68.991 ± 2.40	**0.004**
RP	62.533 ± 2.32	54.177 ± 2.11	**0.009**
BP	64.112 ± 2.17	59.716 ± 2.80	**0.217**
GH	56.530 ± 2.10	50.965 ± 2.65	**0.031**
VT	50.113 ± 2.19	46.448 ± 2.58	**0.281**
SF	66.784 ± 1.15	58.567 ± 1.98	**0.006**
RE	63.554 ± 1.67	57.114 ± 2.09	**0.018**
MH	71.754 ± 2.35	65.541 ± 2.52	**0.041**
PCS	53.157 ± 1.39	46.85 ± 1.12	**0.006**
MCS	48.918 ± 1.30	44.176 ± 1.11	**0.007**

Values are expressed as mean +standard error mean (SEM); P value ≤ 0.05 was considered as statistically significant *BP* bodily pain*, GH* general health*, MCS* mental component score, *MetS* Metabolic Syndrome*, MH* mental health*, PCS* physical component score*, PF* physical functioning, *RE* role emotional*, RP* role physical, *SF* social function, *VT* vitality.

**Fig. 2:**
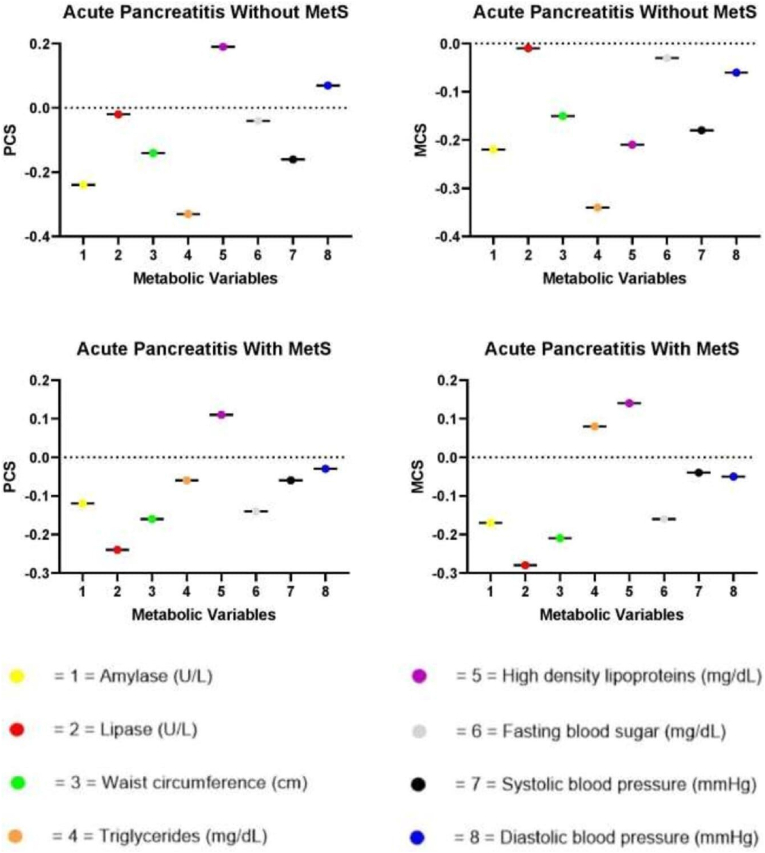
Clinical variables univariate correlation with the eight domains of SF-36 in patients with acute pancreatitis (AP) with and without metabolic syndrome (MetS).

The Univariate Pearson correlation (Table 4 and corresponding Fig. 2.) depict for the metabolic components and 8-SF36 domains corresponding to physical and mental health scores in both groups. In subjects with AP and without MetS, metabolic variables (Amylase, Lipase, WC, TGs, FBG, and SBP) exhibited a statistically significant (P ≤ 0.05) inverse relationship, whereas HDL and DBP showed a positive relationship with the PCS. Furthermore, these patients all metabolic parameters (Amylase, Lipase, WC, TGs, HDL, FBG, SBP and DBP) exhibited a significant inverse link with the MCS. In individuals with the AP with MetS metabolic variables (Amylase, Lipase, WC, TGs, HDL, blood glucose level fasting, blood pressure) had aninverse relationship with the PCS and positive correlation with HDL while Amylase, Lipase, WC, FBG, SBP, and DBP levels were negatively along with TGs and HDL were positively correlated with MCS. All the PCS and MCS values in both AP patients with and without AP correlation values were statistically significant (P ≤ 0.05) with the metabolic markers.

**Table 4:** Univariate correlation of metabolic variables with the eight domains of SF-36 in patients with acute pancreatitis (AP) with and without metabolic syndrome (MetS).

Clinical Variables		Acute Pancreatitis			
		**Without MetS**		**With MetS**	
		**Health Domains of SF-36**		**Health Domains of SF-36**	
		**PCS**	**MCS**	**PCS**	**MCS**
Amylase	r	−0.24	−0.22	−0.12	−0.17
	*P value*	**0.03**	**0.03**	**0.05**	**0.07**
Lipase	r	−0.02	−0.01	−0.24	−0.28
	*P value*	**0.04**	**0.05**	**0.003**	**0.02**
Waist circumference (cm)	r	−0.14	−0.15	−0.16	−0.21
	*P value*	**0.02**	**0.001**	**0.001**	**0.04**
Triglycerides (mg/dL)	r	−0.33	−0.34	−0.06	0.08
	*P value*	**0.007**	**0.008**	**0.4**	**0.3**
High density lipoproteins (mg/dL)	r	0.19	−0.21	0.11	0.14
	*P value*	**0.003**	**0.3**	**0.4**	**0.2**
Fasting blood sugar (mg/dL)	r	−0.04	−0.03	−0.14	−0.16
	*P value*	**0.02**	**0.009**	**0.3**	**0.4**
Systolic blood pressure (mmHg)	r	−0.16	−0.18	−0.06	−0.04
	*P value*	**0.01**	**0.003**	**0.006**	**0.04**
Diastolic blood pressure (mmHg)	r	0.07	−0.06	−0.03	−0.05
	*P value*	**0.001**	**0.04**	**0.02**	**0.02**

*MetS* metabolic syndrome, *r* correlation coefficient, *SF-36* Short Form-36.

## 4 Discussion

MetS has been identified as a prevalent clinical condition that is caused by a bunch of numerous metabolic disorders and is linked to higher death rates [21,22]. The association between MetS's influences on HRQoL in patients suffering with AP is unclear, and there is a dearth of studies on the subject [4]. Niknam et al., in 2020 evaluated 214 patients with AP and concluded that hyperglycemia and hypertriglyceridemia which are the components of MetS showed an impact of increasing severity of AP [23]. In this prospective observational study subjects showed a major connection between the presence of AP with MetS and worsening of physical and mental HRQOL as compared to AP without MetS. Within the same context another clinical study performed by Olesen et al., in 2021 also concluded that older age patients in AP patients with high TGs level were more in number and had poor HRQoL [24]. Educational status also plays a major role in patients and in the present study also it was demonstrated that patients suffering with AP with Mets had no formal education that patients with AP without MetS. In favor to the present study another clinical study performed by Liang et al., in 2021 concluded that education status of the patients as well as their mother's play a major role and had high incidence of causing MetS and poor HRQoL [25]. Subjects with AP with MetS were more in number compared to other group for those who were dependent on tobacco and alcohol consumption. Similarly other clinical studies also concluded that subjects on tobacco and alcohol consumption had greater prevalence and impact on MetS and poor HRQoL [26,27].

AP males with MetS had high mean BMI and WHR as compared to those without MetS and similar was seen in females suffering from AP with MetS had high BMI and WHR as compared to those without MetS and both were found to be statistically significant. AP patients with MetS had substantially higher Amylase, Lipase, WC, TGs, FBG, SBP, DBP, and lower HDL cholesterol than AP patients without MetS in this research. In general, patients with AP with MetS had considerably poorer SF-36 PCS and MCS evaluations than those with AP without MetS. Individuals with AP with MetS had a marked reduction in all SF-36 subscales, except BP and VT, when compared to patients with AP without MetS. Vooturi and Jayalakshmi in 2020 also demonstrated in their clinical study that subjects with epilepsy and MetS had poor MCS and PCS components than subjects with epilepsy without MetS [28]. Also, another clinical study performed by Lin et al., in 2021 in same context concluded that community dwelling adults with MetS had lower mental and physical health scores than those without MetS [29]. These metabolic markers were compared for the correlation with physical and mental health scores in patients of AP without and with MetS. In the present study Amylase, Lipase, WC, FBG and SBP were found to show negative correlation with MCS and PCS in both groups of AP with and without MetS. TGs and DBP were also negatively correlated with all the mental and physical components in both the groups except TGs showed positive relationship with mental health score in AP with MetS and DBP showed positive correlation with PCS in AP without MetS. In contrast, lipoproteins showed positive relationship with all the components in both groups of AP except showing negative correlation with MCS in AP without MetS. In previous investigations, the SF-36 was used to measure HRQOL in subjects with MetS [[30], [31], [32]].

Various Indian research have evaluated HRQOL using general assessment and tools for various gastroenterological disorders in patients, and so on, but none have assumed that linked metabolic variables decrease QOL in patients with AP. This is the first study from India to indicate a link between MetS and the HRQoL of AP. Our study's key strength was a thorough investigation of the MetS components, as well as their influence, correlation and association with HRQoL patients, despite its small sample size.

## 5 Conclusion

In conclusion, comparing subjects with AP and associated MetS to subjects with AP without associated MetS, the current study discovered a strong link between decreased HRQOL in patients with MetS. Patients with AP, on the other hand, are constantly influenced due to disorder's hardships and daily negligence, along with other metabolic variables such as hyperglycemia, insulin resistance, cardiovascular disorders alone or together, puts patients at an even greater physical and psychological risk, due to which subjects with metabolic disorders experience a faster deteriorating impact on health domains, which is why patients with MetS experience a much faster pain. However, because the mechanism behind these relationships is uncertain, more study is needed to fully understand the link between MetS and AP. The thorough assessment of the MetS components, the severity of AP, and the optimal follow-up of all included patients were the key strengths of our research, despite the study's small sample size being a limitation. As a result, it's essential to analyze the wellbeing of people not merely in perspective of lifesaving through different medicines, but also in terms of improving quality of life.

## Ethical approval

The study was performed conforming to the Helsinki declaration of 1975, as revised in 2000 and 2008 concerning human and animal rights and the authors followed the policy concerning informed consent. The study was approved by the Chitkara University Institutional Human Ethical Committee and has been carried out in accordance with the principles of the Declaration of Helsinki as revised in 2000. Written informed consent for participation in the study was taken from each patient or their relative.

## Source of funding

None.

## Author contributions

Ojus Sardana: Conceptualization, Data curation, Writing-Original draft preparation, Writing- Reviewing and Editing. Pratima Kumari: Conceptualization, Data curation, Writing-Original draft preparation, Writing- Reviewing and Editing. Ravinder Singh: Data curation, Writing-Original draft preparation, Writing- Reviewing and Editing. Hitesh Chopra: Data curation, Writing-Original draft preparation, Writing- Reviewing and Editing. Talha Bin Emran: Conceptualization, Writing-Reviewing and Editing, Visualization.

## Research registration number (UIN)

Not applicable.

## Guarantor

Talha Bin Emran, Ph.D., Associate Professor, Department of Pharmacy, BGC Trust University Bangladesh, Chittagong 4381, Bangladesh. T: +88-030-3356193, Fax: +88-031-2550224, Cell: +88-01819-942214. https://orcid.org/0000-0003-3188-2272. E-mail: talhabmb@bgctub.ac.bd.

## Trial registry number

Not applicable.

## Consent

Not applicable.

## Declaration of competing interest

None.
